# Advances in NO_2_ sensing with individual single-walled carbon nanotube transistors

**DOI:** 10.3762/bjnano.5.227

**Published:** 2014-11-20

**Authors:** Kiran Chikkadi, Matthias Muoth, Cosmin Roman, Miroslav Haluska, Christofer Hierold

**Affiliations:** 1 Micro and Nanosystems, Department of Mechanical and Process Engineering, ETH Zurich, Switzerland

**Keywords:** carbon nanotube field-effect transistors (CNFETs), cross-sensitivity, functionalization, gas sensors, hysteresis, low power, selectivity, self-heating, single walled carbon nanotubes

## Abstract

The charge carrier transport in carbon nanotubes is highly sensitive to certain molecules attached to their surface. This property has generated interest for their application in sensing gases, chemicals and biomolecules. With over a decade of research, a clearer picture of the interactions between the carbon nanotube and its surroundings has been achieved. In this review, we intend to summarize the current knowledge on this topic, focusing not only on the effect of adsorbates but also the effect of dielectric charge traps on the electrical transport in single-walled carbon nanotube transistors that are to be used in sensing applications. Recently, contact-passivated, open-channel individual single-walled carbon nanotube field-effect transistors have been shown to be operational at room temperature with ultra-low power consumption. Sensor recovery within minutes through UV illumination or self-heating has been shown. Improvements in fabrication processes aimed at reducing the impact of charge traps have reduced the hysteresis, drift and low-frequency noise in carbon nanotube transistors. While open challenges such as large-scale fabrication, selectivity tuning and noise reduction still remain, these results demonstrate considerable progress in transforming the promise of carbon nanotube properties into functional ultra-low power, highly sensitive gas sensors.

## Introduction

New materials often generate entirely new possibilities, pushing the limits of the accepted boundaries of material properties within which engineers operate. The identification of carbon nanotubes (CNTs) [1–4] and later, single-walled nanotubes (SWNTs) [5–6] is one example of this phenomenon. One such exciting prospect for carbon nanotubes is in the field of chemical sensing. First reports of the sensitivity of the electrical characteristics of carbon nanotubes to adsorbed gases were reported by Kong et al. [7] and Collins et al. [8] in 2000. Kong et al. showed that gases such as NO_2_ and NH_3_ caused a large shift of the threshold voltage in carbon nanotube field-effect transistors (CNFETs). SWNTs possess several properties that make them attractive candidates for gas sensors. They have all their atoms on the surface, endowing them with the highest specific surface area possible together with graphene. Therefore, all the carbon atoms in the nanotube can, in principle, interact with the analyte gas, while simultaneously supporting charge transport in the device. Thus, adsorbates and electrostatic charges and dipoles close to the nanotube can greatly impact charge transport. At the same time, the carbon nanotube lattice is held together by strong sp^2^ C–C bonds, which provide the necessary chemical stability to the carbon nanotube.

The sensitivity of SWNTs towards NO_2_ at ambient temperatures, as reported by Kong et al. [7] is particularly interesting. NO_2_ is a well-known toxic gas and air pollutant and monitoring its concentration is crucial for applications such as air quality monitoring. Leveraging the NO_2_ sensitivity of carbon nanotubes to build highly sensitive, low-power gas sensors is therefore not only of academic, but also of great commercial interest. As such, we will focus on the NO_2_-SWNT system, as it is one of the most extensively discussed in literature.

Several device architectures have been used for fabricating NO_2_ sensors by using carbon nanotubes – as individual elements in the transistor channel [7,9–11], as low density networks [12–15] or as forests [16]. SWNT networks and forests have been shown to be effective for gas sensing, and can be fabricated efficiently on a large scale at low cost. However, the sensing behavior of nanotube networks is very complex due to the large variety of available adsorption sites, the presence of grooves and junctions between nanotubes, photoresist and other processing residues, interfaces with the substrate and tube-metal junctions. Their role in carrier transport and their response to analyte exposure can be diverse and difficult to reproduce. To circumvent this difficulty, empirical or phenomenological models are typically employed to evaluate the gas sensor response in SWNT network sensors. Furthermore, there is recent evidence that the electronic nature of the nanotubes (semiconducting or metallic) and the fabrication processes typically used to fabricate these devices play a substantial role in device performance [17] and the ensuing sensor response [18]. Thus, the behavior of a random ensemble of unsorted nanotubes cannot be used to elucidate the interactions between the analyte molecules and the surface of the nanotube without the interference of the aforementioned effects. Individual SWNT sensors, on the other hand, have a much simpler structure which eases understanding and reproducibility.

One concern for NO_2_ sensing with carbon nanotubes is the selectivity of nanotubes to NO_2_, i.e., their ability to distinguish NO_2_ in the presence of other interfering analytes. For individual, pristine single-walled carbon nanotubes, calculations and experiments have shown that the interactions with other gases present in the atmosphere, such as N_2_, H_2_, CO, H_2_O and CO_2_ are negligible [19]. However, nanotubes are sensitive to other interfering pollutants, such as SO_2_ [20], NH_3_ [12] and O_3_ [21]. Therefore, the task of enhancing the selectivity of SWNT sensors to NO_2_ must also be considered. Employing arrays of such individual SWNT sensors may be a viable option for enhancing the selectivity, sensitivity and robustness of SWNT gas sensor products.

In this review, we will focus on individual nanotube gas sensors and collate the theoretical and experimental achievements of the past few years in understanding the interaction of individual, single-walled carbon nanotubes with their environment. First, a summary of the effects of adsorbate molecules on electronic transport is presented, after which the effect of nearby charges and dipoles on the nanotube carrier transport, which constitutes the major challenge for the stability, resolution and drift of these sensors, is discussed. The list of cited works is by no means exhaustive, but we attempt to highlight the pioneering reports showing relevant information to individual single-walled carbon nanotubes for NO_2_ sensing.

## Review

### Individual-nanotube transistors for gas sensing

**Figure 1:**
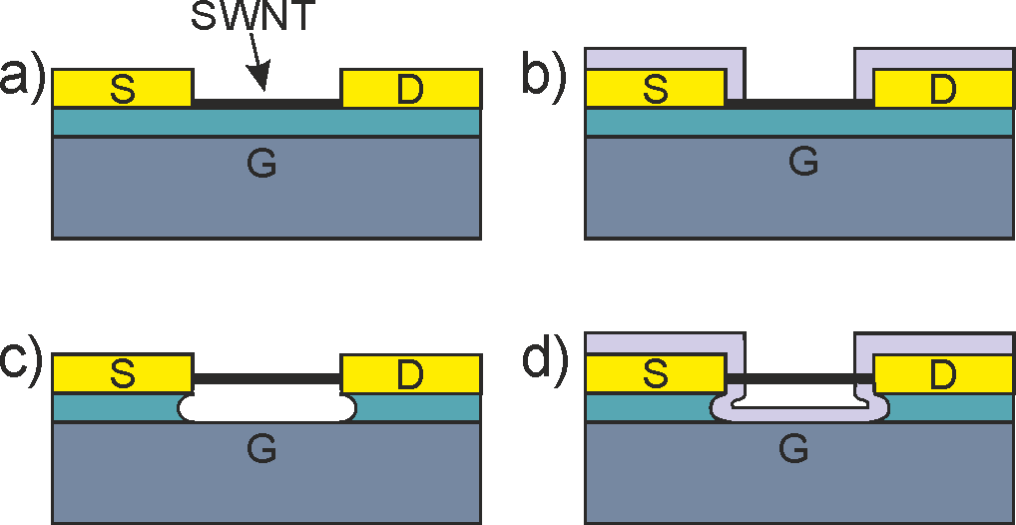
Examples of different back-gated device architectures employed for carbon nanotube field-effect transistor gas sensors. a) On-substrate, unpassivated devices, where the entire device is exposed to the analyte. The substrate acts as the back gate, and the nanotube lies on a dielectric which is deposited on top of this electrode [7,14]. b) Contact-passivated CNFETs. In these devices, only the carbon nanotube is exposed to the gas, while the contacts are passivated against a gas exposure. Passivation layers may include polymers [22] or oxides [9–10]. c) Suspended, unpassivated devices. In this case, the SWNT can be released after fabrication [23–24] the SWNT is either placed [25] or grown [26–28] onto prefabricated electrodes. d) Contact-passivated, suspended-SWNT transistors that are fabricated either through post-fabrication release [29] or using selective film nucleation [30].

The typical individual-SWNT gas sensor is a 3-terminal device, in which the nanotube is contacted by a source and a drain electrode and a current flowing through it is measured. Depending on the work function difference between the metal and the nanotube, a Schottky barrier is commonly formed at both contacts. The channel conductivity is controlled by a gate electrode that is separated from the nanotube channel by a gate dielectric. In Schottky barrier field-effect transistors, the effect of the gate voltage is to change the width of the Schottky barrier, which controls the current through the device. Carbon nanotube transistors often operate as Schottky-barrier field-effect transistors because the back-gated architecture that is typically employed for CNFET gas sensors covers the entire channel and overlaps the source and drain contacts. Top-gate architectures, which have a closer resemblance to CMOS architectures, are less common for gas sensors due to the need for an exposed channel. Side-gate configurations are not typically used due to their poor gate coupling. Variants of this basic architecture, with suspended or on-substrate tubes, and passivated or unpassivated contacts have been reported (Figure 1).

The conductivity of a CNFET can be influenced by surface adsorbates. In particular, the adsorption of electron-withdrawing gases, such as NO_2_, or electron-donating gases such as NH_3_ can influence the electron transport in the devices by changing the transfer characteristics. However, the devices also react strongly to charges in their vicinity, which leads to problems such as hysteresis and noise. We will examine these effects in detail in the next sections.

### Effect of adsorbate gases

The sensitivity of carbon nanotube devices to adsorbate gases was reported in 2000 by two groups. Collins et al. [8] showed that carbon nanotube mats contacted by metal showed oxygen sensitivity, while Kong et al. [7] showed the sensitivity of individual CNFETs to NO_2_ and NH_3_, and discussed the possible mechanisms behind the device response. They reported that the CNFET responded to gas exposure by showing a large shift of the transfer (*I*_d_–*V*_g_) characteristics, with the direction of the shift dependent on the gas type.

The remarkable feature of the observations by Kong et al. [7] is that the nanotubes were sensitive to gases at ambient temperature. Most solid-state semiconducting gas sensors that operate as chem-FETs require elevated temperatures for operation. Heating is an energy-expensive operation, due to which their application in large numbers or in sensor network nodes is challenging. Carbon nanotubes also appeared to be highly sensitive even without optimization, hinting at their promise for NO_2_ detection.

Several studies, both experimental and theoretical, have been conducted to understand the effect of gas molecules on carbon nanotube devices. Understanding the effect of gases on the CNFET electrical transport is crucial to sensor design and architecture. For a CNFET to be sensitive to a certain gas, the gas molecules must be able to interact and bind to the device surface and produce a change in the electrical transport of the device. A lively debate is prevalent in literature about the sensing mechanisms involved, and both contact-related and channel-related mechanisms have been suggested. For the sake of discussion it is convenient to distinguish the effect of gas adsorption on the two different regions: the contact metal surface and the nanotube surface. In the next few paragraphs, the different sensing mechanisms which may be present in a CNFET sensor are discussed in detail.

### Adsorption on the metal

The work function of a metal is known to be sensitive to the adsorption of gaseous surface species. Methods such as Kelvin probe [31], scanning tunneling spectroscopy, X-ray and ultraviolet photoelectron spectroscopy [32] (XPS and UPS) have been used to understand the mechanism of this work function change. For polar molecules such as NO_2_, a dipole layer is expected to be formed at the surface of the metal, thereby modifying the metal work function [33–34]. In Schottky barrier CNFETs, a change of metal work function means a net change in the metal–nanotube work function difference, which in turn determines the height of the Schottky barrier. For carbon nanotube gas sensors, any sensor response associated with on-metal adsorption is therefore related to a change in the height of the carrier injection barrier at the contacts. As an example, consider the case of a device that has dominant n-type conductivity, i.e., the electron Schottky barrier is smaller than the hole Schottky barrier. The adsorption of NO_2_ can lead to an increase of the metal work function (and thereby an increase in the Schottky barrier height for electrons), leading to a lower current in the n-branch and an increase in the p-branch [35].

On the other hand, a work function change for devices with ohmic contacts will depend on magnitude and direction of the change. For example, if the work function change induces a Schottky barrier in the device (i.e., the device becomes non-ohmic), it could lead to a visible change in the transfer characteristics, resulting in a sensor response. However, if the result of work function change is to turn the Schottky barrier more negative within the same n-type device thought experiment, then no sensor response is expected.

**Figure 2:**
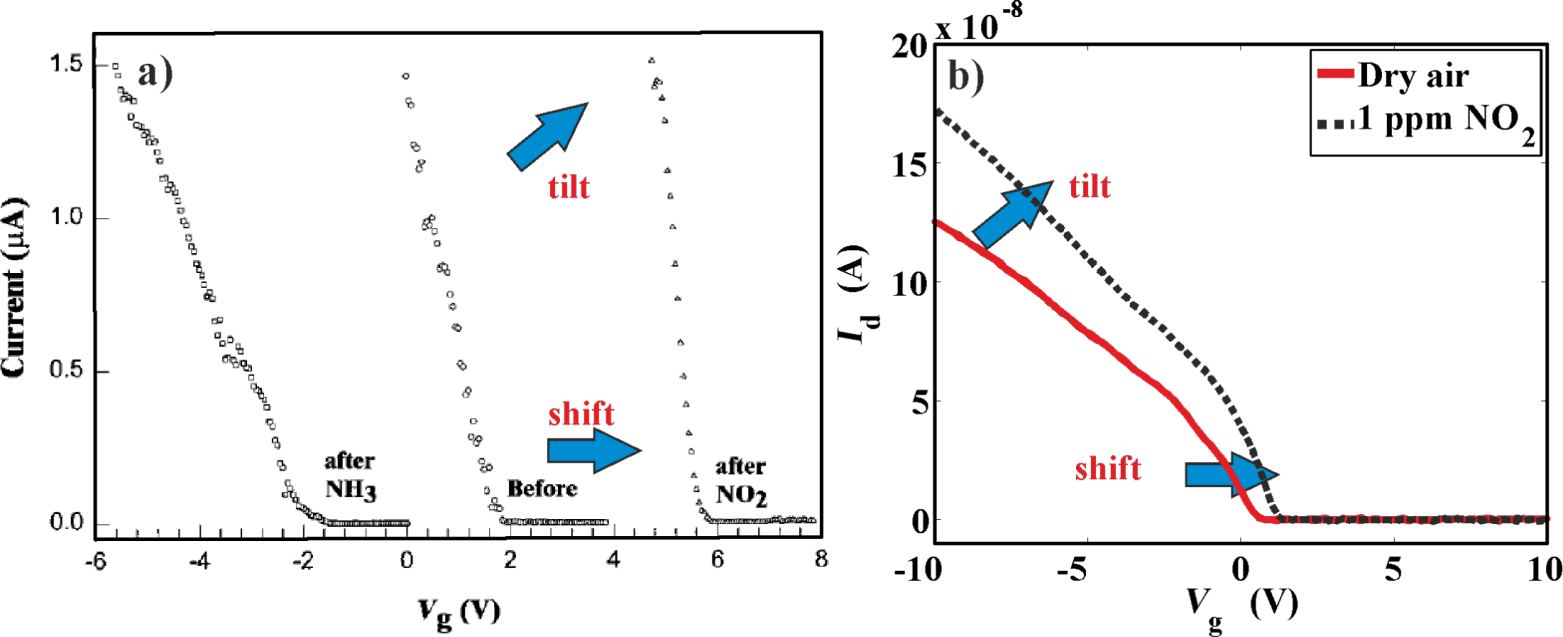
Effects of NO_2_ adsorption on unpassivated nanotube transistors. a) Measurements from Kong et al. [7] show a shift and a tilt of the characteristics, as shown above. In their case, the Ni–Au contacts are likely to have Schottky barriers, due to which the effects may be due to both on-tube and on-metal adsorption. Adapted with permission from [7]. Copyright 2000 American Academy for the Advancement of Sciences. b) The authors have also observed similar effects in [36] for Pd-contacted nanotubes. Adapted with permission from [36]. Copyright 2014 Elsevier.

Sensor response of devices with unpassivated contacts tend to be a tilt of the transfer characteristics (visible in the linear part of the curve) as well as a shift of the threshold voltage; this is often attributed to the effect of adsorption on both the metal (leading to a Schottky barrier change) as well as the nanotube channel (leading to doping) [35], as shown in Figure 2. This is because the devices are unpassivated, and gas adsorption could occur on both the nanotube and the metal surfaces. So far, there have been no successful attempts at measuring the effect of on-metal adsorption separately, due to the fabrication challenges involved in completely passivating the nanotube alone, while leaving the metal unpassivated.

### Adsorption on the nanotube

The nature of interaction between NO_2_ and the nanotube is heavily debated, and the current understanding of the NO_2_–nanotube interaction is far from complete. A brief review of the reports so far will be made before further discussing their effects on the electrical characteristics of the CNFET.

**Figure 3:**
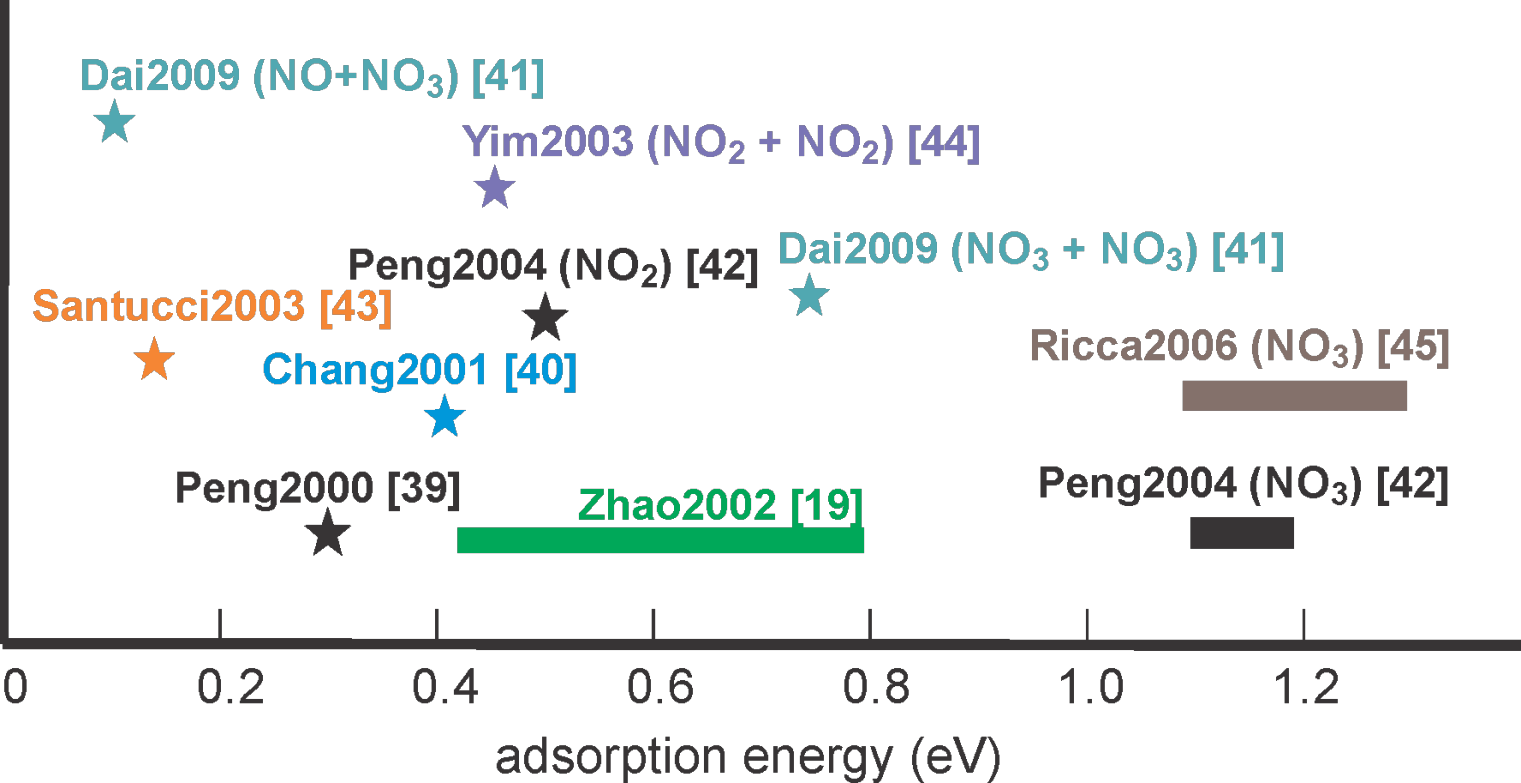
Theoretically calculated adsorption energies for various NO*_x_*–CNT systems. The calculations show widely varying interactions for the systems, which may be partly explained due to the different simulation methods used as well as the different chiralities for the SWNTs.

Several experimental reports have shown that the undisturbed recovery time of a nanotube sensor after exposure to NO_2_ is in the range of several hours [12,30,37–38]. This alone suggests that the desorption barrier for NO_2_ on the nanotube is relatively high – of the order of −1 eV. However, many theoretical calculations (see Figure 3) contend that pure NO_2_ adsorption is physisorption [19,39–43]. Although the first calculations [39–40] suggested a charge transfer upon physisorption, Santucci et al. [43] showed that this charge transfer disappeared when spin-polarized treatment was used in the calculations. Yim et al. [44] have suggested that a pairwise chemisorption of NO_2_ molecules is more favorable than the chemisorption of individual molecules, but the energy of interaction they report is far lower than the observed experimental results as well as theoretical calculations in other reports [41,45].

Another possibility is dissociative adsorption of NO_2_. Goldoni et al. [46–48] performed photoemission spectroscopy measurements of NO_2_ adsorption on purified SWNTs. Their data showed indications suggestive of the formation of NO_3_ and NO on the surface of the nanotube. This is in agreement with the calculations of Peng et al. [42] who reported a strong binding energy for NO_3_ on the nanotube. However, it has since been pointed out that the methods used in this paper tend to overestimate the binding energy [41,45] and charge transfer. Dai et al. [41] found that chemisorption of two NO_3_ molecules, on the other hand, is found to be favorable, with an adsorption energy (for two molecules) between −1.4 and 1.9 eV. On the other hand, the results from Ellison et al. [49] by using temperature-programmed desorption (TPD) and Fourier-transform infrared (FTIR) spectroscopy disagree with this suggestion, as they found no evidence for chemisorption. In addition, these studies also suggest a strong dependence of binding energy on the nanotube curvature, but a systematic experimental investigation of this dependence has not yet been performed.

There are also suggestions that the presence of an electric field or charges on the nanotube may have a strong influence on the binding energy and charge transfer of NO_2_ [50]. Although there have not been any direct studies with NO_2_, Chen et al. [51] calculated the binding energy of water molecules on an SWNT surface and found that it increased from 0.03 eV at zero electric field to 0.6 eV at an electric field of 0.33 V/Å. For comparison, the electric field from the gate in the vicinity of the nanotube in typical CNFET gas sensors can be around 0.1 V/Å [7,52]. As such, the effect of an electric field on the binding energy of NO_2_ on a SWNT remains poorly understood.

A further possibility is that interfaces between the nanotube and the substrate provide more favorable sites for adsorption, where the NO_2_ molecules may have a higher binding energy. However, suspended nanotubes have also been shown to be sensitive to NO_2_ [30,53], and the recovery time even in the absence of a substrate appears to be comparable. This suggests that the nanotube is capable of responding to NO_2_ without the assistance of a substrate.

More recently, evidence has emerged on the possible role of the electronic nature of the nanotube (metallic or semiconducting) in the response of carbon nanotube gas sensors. Ruiz-Soria et al. [18] investigated the NO_2_–SWNT system by utilizing metallicity-sorted, ultrapure carbon nanotubes. They used X-ray photoelectron spectroscopy (XPS) and X-ray absorption spectroscopy (XAS) to investigate the nature of bonding and chemical interaction. Their conclusions, supported by theoretical calculations, are that the process is a charge transfer mediated physisorption. In their studies, both semiconducting and metallic nanotubes showed evidence of NO_2_ physisorption, but the interaction energy was twice as high (as indicated by the C–N peak intensity ratios in their spectroscopic measurements) for metallic nanotubes compared to semiconducting nanotubes. They also found very little NO_3_ and NO formation in their measurements, suggesting that these mechanisms may not be dominant. However, their experiments were based on spectroscopic measurements at low temperature, and they performed no direct electrical measurements, due to which the implications of their conclusions to FET-type devices is not yet completely clear.

**Figure 4:**
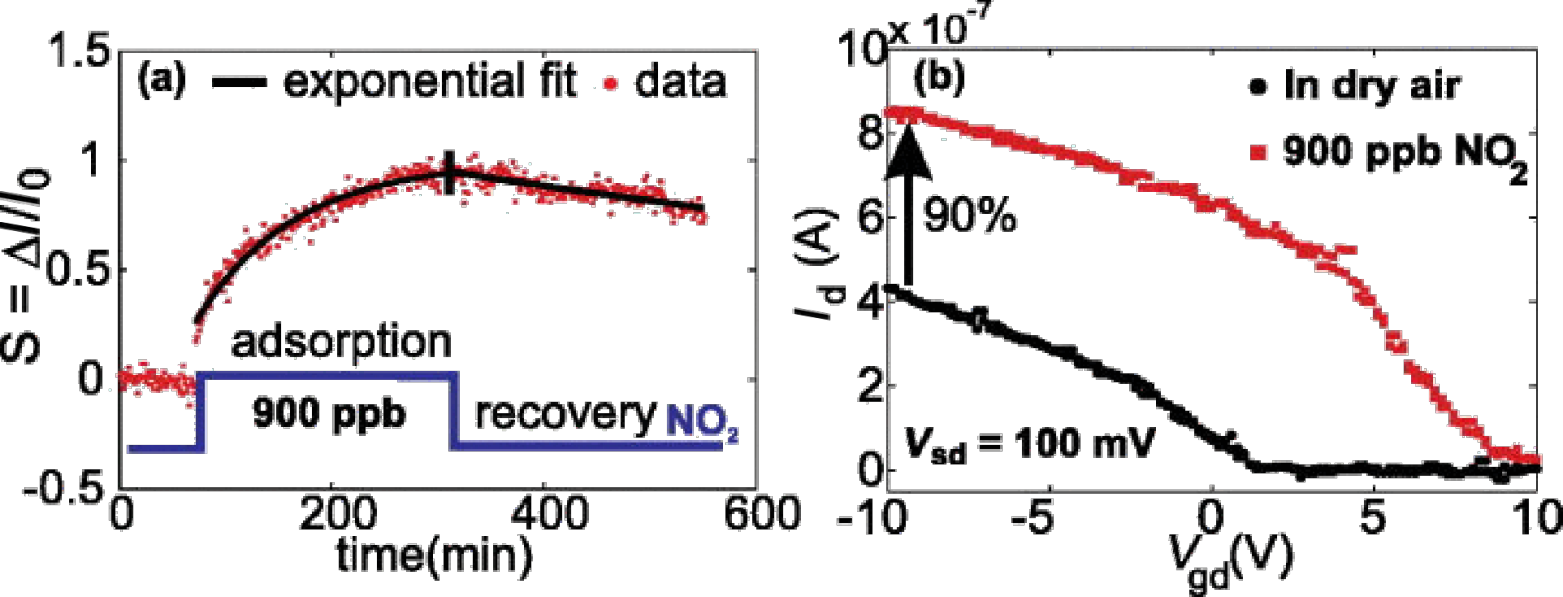
Response of contact-passivated, pristine suspended carbon nanotube gas sensors. a) Transient response of the sensor to an NO_2_ step. The exponential fits to the data are shown. The recovery time is in excess of 12 h, which corresponds to an equivalent desorption barrier of −1.01 eV. b) Transfer characteristics recorded for the same experiment as (a). The black circles were recorded just before the NO_2_ entered the chamber, while the red circles were recorded just before the NO_2_ step ended. Reproduced with permission from [30]. Copyright 2013 AIP Publishing LLC.

In recent studies, we have shown that a single, suspended nanotube with passivated contacts is sensitive to NO_2_ [30], as shown in Figure 4. On the other hand, a fully-passivated device does not respond to gas exposure. This excludes other possibilities such as substrate-assisted adsorption and response from the contacts suggesting that the nanotube itself is indeed sensitive even in the absence of other contributing mechanisms. Furthermore, the long recovery time is observed here as well, suggesting a high desorption barrier for the tube–NO_2_ interaction.

The effect of adsorption on the nanotube on the transistor characteristics can also depend on the device architecture and contact type. Bradley et al. [10] discuss a similar response in contact-passivated CNFETs for NH_3_ sensors, but indicate that this may be related to the screening length in their short-channel FETs. Similar observations are made by Mattmann et al. [9]. On the other hand, Zhang et al. [22] discuss the response of polymer-passivated long-channel FETs which also show an apparent shift of the characteristics. They suggest that the response of the CNFETs to gas can be attributed not to channel doping, but to a change in the Schottky barrier. They further account for the gas diffusion through the polymer membranes and argue that the long response times in their contact-passivated devices are related to the time constants associated with this diffusion, while the unpassivated devices exhibit a faster response. These arguments further suggest that adsorption on the nanotube could indeed generate different responses depending on the contact type. In a Schottky barrier CNFET, if the adsorption on the nanotube close to the barrier changes the screening length, the result on the transfer characteristics could be similar to that produced by a change in the metal work function (the Schottky barrier width changes instead of the height). Adsorption in the bulk of the nanotube causing a doping effect could produce no measurable response in Schottky barrier FETs, as the device conductance is not limited by the channel. On the other hand, in ohmic or near-ohmic contact nanotubes with extremely thin Schottky barriers (e.g., Pd-contacted nanotubes), the modulation of the conductance of the bulk channel could have a more profound effect. This is consistent with the observations made by the authors on Pd-contacted, contact-passivated carbon nanotube transistors [30]. Nevertheless, more detailed experiments on long-channel, ohmic transistors would be necessary to further clarify this question.

Thus, the apparent contradictions and the multitude of sensing mechanisms proposed in literature is a strong indication that the actual sensing mechanism predominant in a device depends not only on the number, quality (i.e., defects [54–56]) and the dimensions of the nanotubes used but also on the device architecture, contact type (Schottky or ohmic), the fabrication process and contact materials. In general, the desorption energies for NO_2_ adsorbed on the metal contacts, defect sites and other interfaces are higher than the interaction between NO_2_ and the nanotube. For low recovery times, therefore, exploiting the NO_2_–SWNT interaction alone, while suppressing other interactions, may be beneficial. In this respect, suspended, contact-passivated devices show great potential.

### Effect of surface functionalization

So far, there have been very few investigations on individual, functionalized SWNTs for NO_2_ sensing. Covalent functionalization on individual SWNTs, in particular, is very challenging because any covalent bonding tends to drastically reduce the conductivity of the SWNT channel by disrupting the sp^2^-C network. Consequently, the conductivity of a single nanotube decreases substantially upon covalent functionalization, making it very challenging to measure its sensor response. Furthermore, fabricating single-tube devices is far more challenging as the yields are lower compared to devices with mats or networks of tubes. As a result, most investigations on functionalized nanotubes involve networks [14–15,37] or forests of nanotubes [16,57].

Non-covalent functionalization, such as metal or polymer functionalization, has been extensively studied. For example, Penza et al. [16] have shown that the sensitivity of multiwalled nanotubes to NO_2_ is enhanced by the use of Pt nanoparticles. However, a comparable study on individual SWNT FETs, for which the mechanisms may be different, is not available. On the other hand, polyethyleneimine (PEI) functionalization for NO_2_ sensing has been shown to dramatically enhance the response of SWNT sensors [14]. There, the authors argue that the main mechanism of sensitivity enhancement is the increased sticking coefficient (by two orders of magnitude) compared to pristine nanotube devices that were also reported by the same group elsewhere [42]. This has the effect of increasing the sensitivity of the nanotube–PEI composite which makes it sensitive to NO_2_ concentrations as low as 100 parts per trillion (ppt). As a comparison, the lowest measured detection limit for unfunctionalized individual SWNTs for NO_2_ is about 5 ppb [13]. Therefore, it may be of further interest to investigate functional groups to enhance the sensitivity of individual carbon nanotube devices to NO_2_.

### Gate hysteresis

Carbon nanotube transistors are well-known for their sensitivity to the charges in their environment. Several detailed investigations have shown that gate hysteresis is an important problem for the stability of the readout in carbon nanotube transistors and gas sensors based on them. When a bi-directional gate sweep is performed, it is often observed that the forward and reverse sweeps are not concurrent, and the measured effect can often be over 50% of the gate sweep range. For gas sensors, this effect can be problematic due to the instability of the device current that arises from the drift and fluctuations associated with this hysteresis. For instance, a device biased at a constant gate voltage often drifts in time, thereby interfering with the gas sensitivity of the sensor.

**Figure 5:**
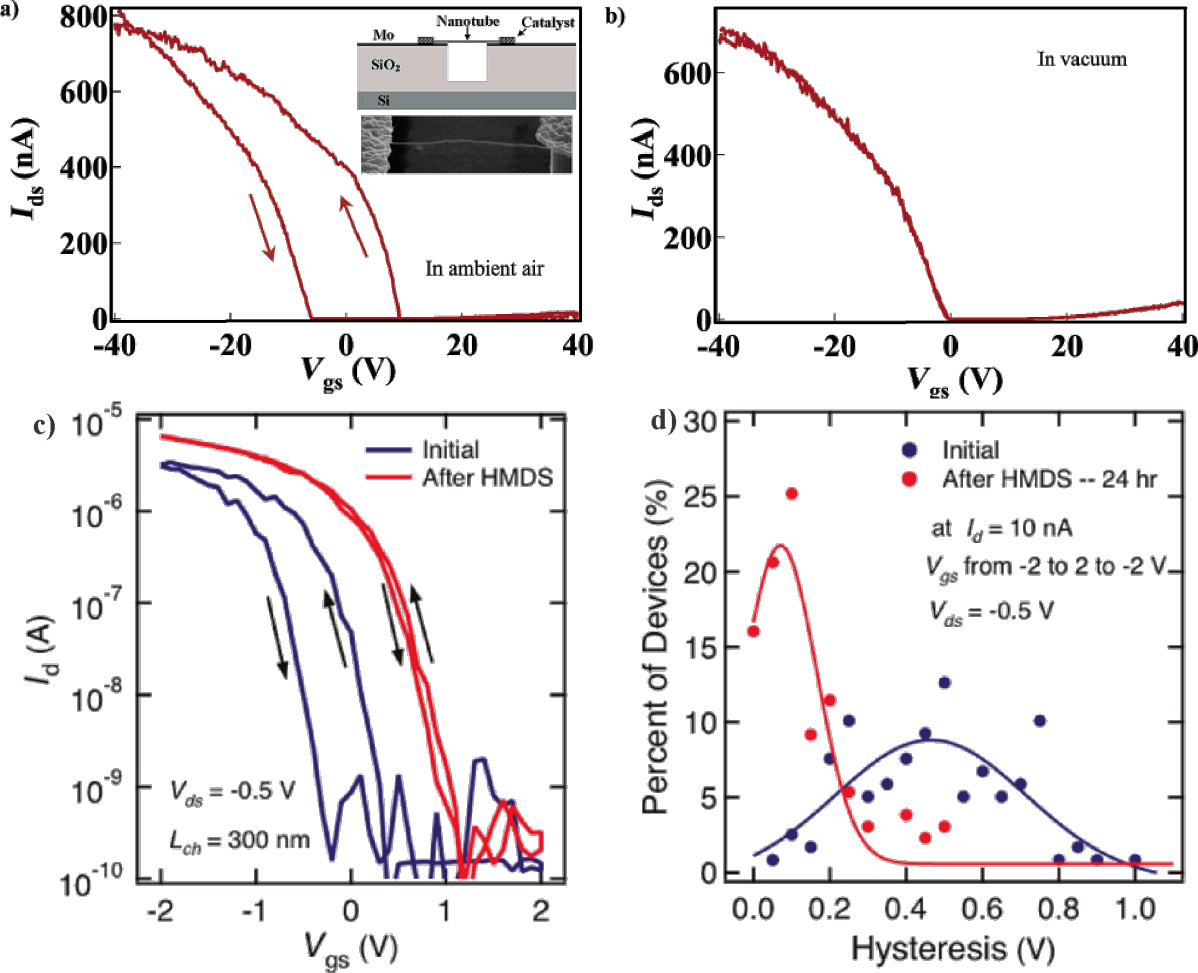
Hysteretic effects in carbon nanotubes lying on a substrate, as shown by Kim et al. [58] (a, b): a) A CNFET partially suspended on SiO_2_, showing hysteresis in ambient air. b) The same device after annealing in vacuum, thereby eliminating the water molecules on the substrate. Reproduced with permission from [58]. Copyright 2013 American Chemical Society. Suppression of hysteresis by surface treatment with an HMDS layer, as shown by Franklin et al. [59] (c, d): c) Transfer characteristics of devices on a substrate, before and after HMDS treatment. d) After 24 h treatment with HMDS, the hysteresis nearly vanishes, as visible from the histograms of the hysteresis width. Reproduced with permission from [59]. Copyright 2012 American Chemical Society.

The source of the hysteresis is attributed to several mechanisms, depending on the device architecture. Kim et al. [58] discuss the effect of water adsorption on substrate-bound carbon nanotube devices, lying on SiO_2_ substrates (Figure 5a and Figure 5b). Due to the hydrophilic surface chemistry of SiO_2_, a thin water layer is present close to the nanotube. These water molecules then act as charge traps that screen the gate voltage and create a field-dependent hysteresis in the transfer characteristics. They further show that annealing in vacuum followed by passivation can drastically reduce the amount of hysteresis, due to the removal of the water species. Franklin et al. [59] have recently shown that passivating the substrate with HMDS is highly effective in reducing hysteresis. In their work, the hysteresis decreases gradually with passivation duration, achieving complete passivation after 24 h and reducing the hysteresis by 83% (Figure 5c and Figure 5d). From these studies, the contribution of water to hysteresis appears to be substantial when present in combination with a hydrophilic substrate.

Other investigations [9,60] showed that even with vacuum annealing and passivation with Al_2_O_3_, although the hysteresis decreases, it does not completely disappear. This suggests other mechanisms at play, such as charge trapping in the oxide and the dielectrics surrounding the nanotube. Robert-Peillard et al. [61] developed a model to qualitatively describe this phenomenon by using Fowler–Nordheim-type behavior for charge trapping by tunneling through the oxide layer. Although this model qualitatively describes the behavior of such devices, it is currently unclear if it reflects the underlying physics of gate hysteresis. More recent investigations have discussed gate hysteresis by using the Schottky–Reed–Hall framework [62].

**Figure 6:**
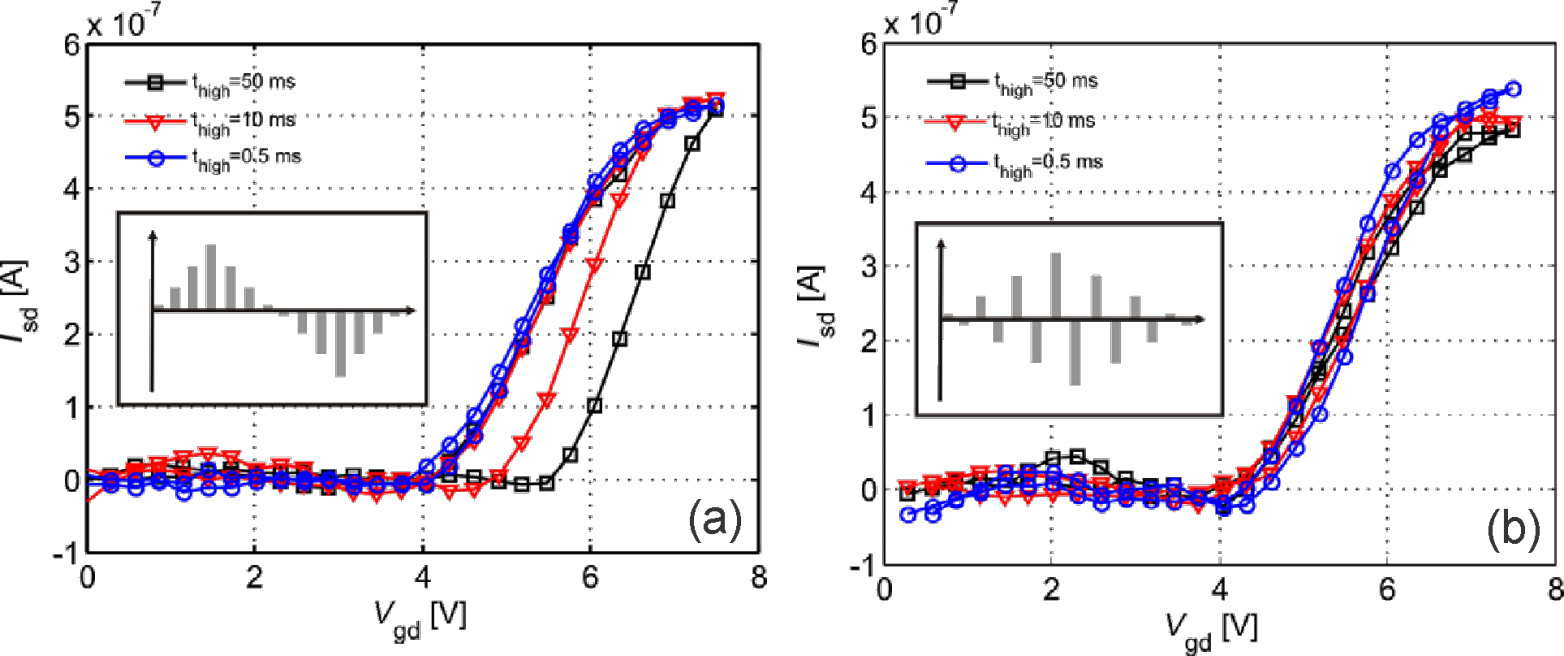
Pulsed gate sweep strategies to eliminate hysteresis in CNFETs [64]. a) Pulsed (p++) gate sweeps show a gradual decrease in the hysteresis width depending on the pulse duration. b) Pulsed (p+-) sweeps with alternating positive and negative pulses show an effective suppression of hysteresis even at high pulse widths. Reproduced with permission from [64]. Copyright 2010 AIP Publishing LLC.

Several methods to minimize hysteresis have been proposed. For unpassivated devices, methods suggested previously, such as exposure to HMDS or other hydrophobic coatings, as well as vacuum annealing are known to reduce hysteresis. Alternatively, Lin et al. [63] showed that by performing gate sweeps in the form of short pulses instead of quasi-static sweeps, hysteresis could be reduced. By pulsing the gate voltage, the duration in which trapping can occur is reduced, and the charges have time to de-trap in the time between gate pulses, thereby reducing the induced screening. Building on this idea, Mattmann et al. [64–65] showed that by applying alternating gate pulses, i.e, every positive gate pulse is followed by a negative gate pulse, hysteresis could be completely suppressed, as the charges trapped in the positive pulse were immediately de-trapped in the subsequent negative gate pulse (see Figure 6). Nonetheless, pulsed measurement schemes are complex and challenging to implement in sensor readout electronics.

If charge trapping in the nanotube surroundings is the primary cause of hysteresis, eliminating these sources, for example by using a protective layer to avoid resist contamination on the SWNT, could be effective in reducing hysteresis [17,66]. By using a protective layer that can later be removed selectively, it is possible to improve the contact resistance as well as reduce the hysteresis simultaneously, as shown by Khamis et al. [66] with organic layers, and Liu et al. [17] with an aluminium oxide layer.

**Figure 7:**
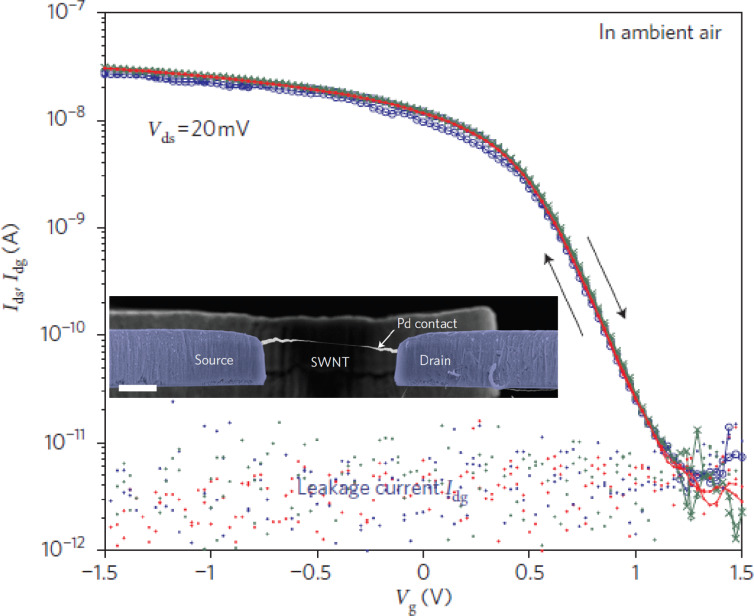
Hysteresis-free transistors using ultraclean, suspended carbon nanotubes. Gate sweeps for a suspended transistor fabricated using on-chip shadow-masking and ultraclean nanotubes shows no measurable hysteresis even in humid air. Reproduced with permission from [28]. Copyright 2010 Macmillan Publishers Ltd.

Another possibility is to eliminate dielectrics altogether, for example by suspending the nanotube, while simultaneously keeping the SWNT surface contamination-free [28]. Initial reports on suspended nanotube devices which were fabricated through top-down lithography followed by release failed to show this behavior, as substantial hysteresis was still present in such devices. However, further work showed that preventing resist residues and wet chemical processing was just as important, as common fabrication process steps created undesirable surface modifications on the SWNT. Reports [26,67] have shown that suspending the nanotube often results in a reduction of hysteresis. Furthermore, Muoth et al. [25,28,68] have shown that in the absence of wet chemical processing, dielectrics or resist residues on the nanotube, hysteresis completely disappears even in ambient air (see Figure 7), further suggesting that the water-related hysteresis mechanisms observed in previous studies are present only when the nanotube is in contact with a substrate such as SiO_2_.

### Drift and low-frequency noise

**Figure 8:**
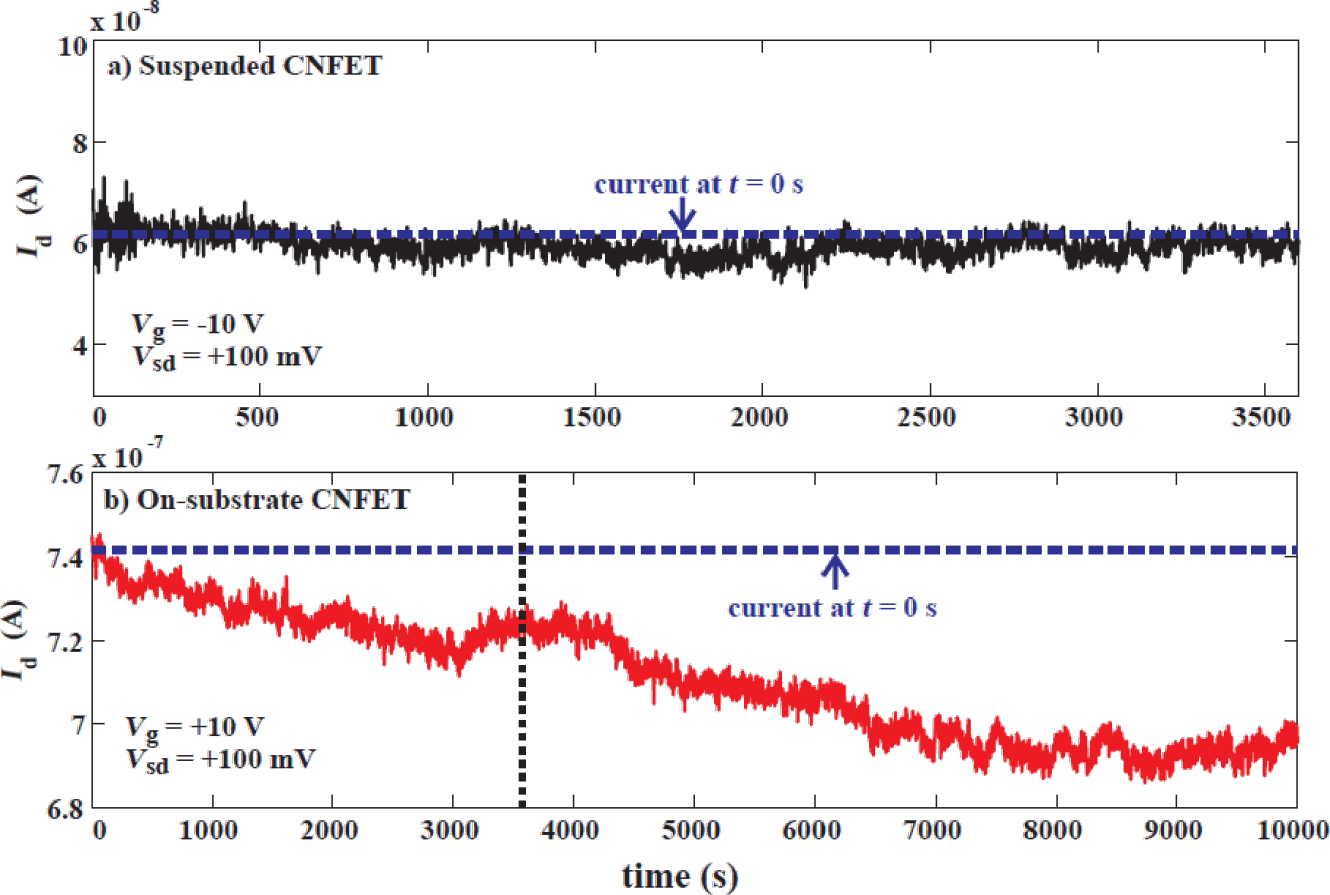
Drift suppression in suspended carbon nanotubes a) suspended carbon nanotube transistor and b) substrate-bound carbon nanotube transistor under constant bias. Over the same time span (marked by the vertical dotted line), the on-substrate transistor shows a 4% drop in current compared to the suspended transistor. The on-substrate transistor continues to decay beyond this point, decaying by about 10% over 10000 s of measurement. Reproduced with permission from [36]. Copyright 2014 Elsevier.

Drift of a sensor may have several origins, related to the ageing of the device, changes in materials properties over time, or ultra-low frequency noise. For example, if a substrate-bound CNFET sensor is measured under constant bias, the signal tends to drift monotonically over several hours due to the continuous accumulation of charges in the vicinity of the nanotube. In this respect, this source of drift is closely related to the hysteresis in the device, since they have common origins in charge trapping in the vicinity of the nanotube. This effect could be minimized by using suspended device architectures, as the authors have shown in [36]. Comparing a suspended, ultra-clean carbon nanotube transistor and a substrate-bound sensor (Figure 8), we see that the drift is strongly suppressed in the suspended transistor, due to the absence of charge traps in the vicinity.

Another closely-related aspect is the current fluctuation that is observed over very long periods of time, which can also contribute towards sensor drift. This ultra-low frequency noise in CNFETs has a 1/*f* power spectral density. In the range of a few Hz to a few μHz, where sensor readout is commonly performed, this noise can be a significant roadblock to sensor performance. Collins et al. [69] first reported the presence of significant 1/*f* noise in carbon nanotube nanodevices. In 2006, Lin et al. [70] studied the gate–voltage dependence of this noise, which was later explained by Tersoff [71] by introducing the so-called charge-noise model. According to this model, the injection of charges into traps near the Schottky barrier can lead to changes in the width of the barrier, which is then manifested as a change in the current through the transistor that is determined by the local gate potential. As an extension, the model contains another term to model 1/*f*-fluctuations that do not depend on the gate voltage. The noise amplitude *A* is then modeled dependent on gate voltage *V*_g_ and drain current *I*_d_ as:

[1]
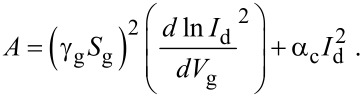


Here γ_g_ is related to the oxide quality and is influenced by the charge traps available around the nanotube, *S*_g_ is proportional to the subthreshold slope and α_c_ is the noise parameter independent of the gate voltage. This model was developed for ballistic carbon nanotube transistors, but has found validity in long-channel Schottky-barrier CNFETs as well. For example, Männik et al. [72] and Helbling et al. [73] showed that these low-frequency fluctuations in carbon nanotube sensors on a substrate could also be explained by the extended charge-noise model, and studied the gate-voltage dependence to identify the ideal quiescent point for operating these sensors.

**Figure 9:**
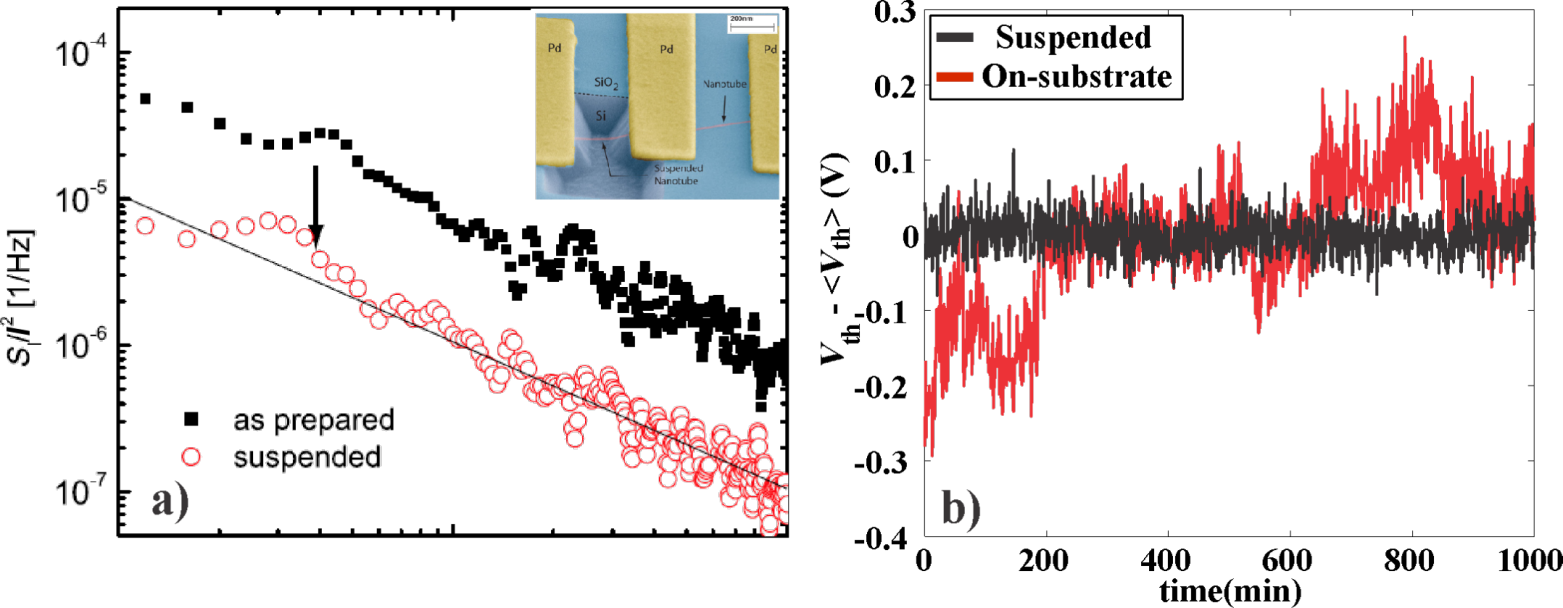
Reduction of 1/*f* noise in suspended CNFETs. a) The results from Lin et al. [23] show a 10-fold decrease in noise amplitude for a suspended transistor compared to a on-substrate transistor on the same nanotube. Reproduced with permission from [23]. Copyright 2007 IOP publishing. b) We have shown ultraclean, suspended transistors that exhibit remarkably low threshold-voltage fluctuations compared to on-substrate transistors [36]. Reproduced with permission from [36]. Copyright 2014 Elsevier.

Lin et al. [23] also showed by comparing suspended and on-substrate devices on the same carbon nanotube, that the 1/*f*-noise in the suspended sections decreased by an order of magnitude due to the elimination of the oxide layer (Figure 9a). Building on this concept, the authors have recently shown in [36] that combined with the use of ultraclean fabrication processes for gas sensors, suspended gas sensors showed a 9-fold improvement in the signal-to-noise ratio compared to on-substrate sensors as well as improved stability of the threshold voltage (Figure 9b). This increase in signal-to-noise ratio directly implies improved sensor resolution.

### Recovery

For a sensor to be functional, it must be fully recoverable after each cycle of exposure to gas. For NO_2_ sensing using carbon nanotubes, this is a unique challenge. As discussed earlier, the undisturbed recovery time of nanotubes from NO_2_ exposure is of the order of several hours. This is impractical for sensor applications.

In order to accelerate the recovery of the sensor, energy is supplied to the device in the form of heat or UV radiation. UV light has been used for carbon nanotube networks to recover the sensor [12–13]. However, Chen et al. [13] suggest that their individual-tube devices were not exposed to UV, citing the vulnerability of the single-tube device. Indeed, they also showed a drastic reduction in the conductivity of their SWNT-mat upon exposure to UV radiation. A single nanotube would likely be damaged over repeated UV-cycling for recovery, and therefore this may not be a viable long-term recovery solution.

Kong et al. [7] on the other hand showed that the sensor could be recovered by heating in vacuum for 1 h at 200 °C. Other papers [9] have subsequently shown that heating between 100 °C and 200 °C for between 10 min and 1 h recovers the sensor. Therefore, this approach appears to be a promising method, but it requires the integration of heaters into the device architecture. Heaters generally consume substantial power (around 10–100 mW for microheaters [74–75]), which nullifies the substantial power advantage that is achieved through the room-temperature operation of carbon nanotube gas sensors.

Instead of an external heater, the authors have recently reported on a self-heated, suspended carbon nanotube gas sensor, that can recover from NO_2_ exposure at a power below 3 μW within 10 min. The suspended architecture of the device plays a significant role in minimizing the power consumed by the device, since the isolated nanotube does not have any heat loss paths to the substrate. This ultra-low power consumption for the sensor provides a promising approach to sensor recovery while maintaining the power advantage of the sensors.

### Performance Summary

Table 1 provides a summary of the performance of individual carbon nanotube gas sensors reported so far. In some cases, the numbers are extrapolated from the results reported in the papers, and are appropriately indicated.

**Table 1:** Performance summary of individual or few-tube SWNT-based NO_2_ sensors. Sensors incorporating mats or forests of nanotubes are not considered. PEI: polyethyleneimine. It must be noted that the electrically refreshed gas sensors reported by Chang et al. [76] has been disputed by a subsequent article from Ervin et al. [77] who suggest that hysteretic contributions may be responsible for this effect.

authors	functional material	lowest conc.	response time (min)	recovery time (min)	recovery method

Kong et al. [7]	none	2 ppm	5 at 2 ppm	60	heating to 200 °C
Qi et al. [14]	PEI	0.1 ppb	1–2 at ppb	1–2	UV light (254 nm)
Zhang et al. [22]	none	n.a.	41 at 200 ppm	10	heating at 100 °C
Suehiro et al. [78]	none	0.5 ppm	1^a^	n.a.	UV light
Chang et al. [76]	none	300 ppm	n.a.	few seconds	voltage pulses
Mattmann et al. [65]	none	50 ppb	30	60	heating at 110 °C
Chen et al. [13]	none	5 ppb	30 at 5 ppb	n.a.	n.a.
Chikkadi et al. [11]	none	200 ppb	90 at 900 ppb	10	self-heating

^a^estimated value

## Conclusion

Individual, single-walled CNFETs have long been used as model systems to study interactions between specific analytes and the nanotube, but several advances in fabrication processes as well as understanding of their behavior has enabled the prospect of using individual-tube devices directly in applications. Substantial progress has been made in understanding the source of noise, drift and hysteresis, and techniques have been introduced to counteract them, such as pulsed measurements and suspended device architectures. Furthermore, elimination of process residues and dielectrics leads to a complete suppression of hysteresis and an approximate 9-fold improvement in the noise performance [36]. Suspended devices are also attractive for self-heated, low-power architectures [30].

There are still several challenges to be addressed, particularly in the large-scale fabrication of these devices. Although integration of individual-nanotube devices on a wafer-scale has been achieved with moderate yield, the placement, length and chirality control remain challenging. Additional challenges remain in characterizing these devices in humid environments. Humid environments may be challenging for on-substrate devices owing to the induced hysteresis and noise, but suspended, ultraclean devices are expected to cope better with this problem due to the absence of hysteresis. Furthermore, the effect of functionalization must be studied in more detail on individual, suspended SWNTs in order to gain a better understanding of the mechanisms involved, and reliable techniques must be developed to extend these functionalization techniques on a large scale.

Although the debate on the precise nature of the NO_2_-nanotube binding is not fully settled, experiments have made progress on understanding the nature of this interaction. From a theoretical perspective, the effect of NO_2_ on the electron transport in the SWNT must be explored in more detail, and a hypothesis that consistently explains the observations from several papers must be developed. As it has increasingly grown clear, the cleanliness and type of the nanotube may play a significant role in the sensor response, and a clear link between the theoretical understanding and the experimental result will contribute significantly towards a better design for all gas sensors employing carbon nanotubes as their functional material.

The utility of individual-tube devices, furthermore, is not limited to addressing academic questions regarding sensing mechanisms and probing the limits of the technology. With improving control over fabrication processes and employing arrays of such individually-contacted SWNT devices, either for enhanced selectivity or noise performance, gas sensor products operating at extremely low powers can be envisioned. Compared to nanotube networks employing the same number of nanotubes, a better signal-to-noise ratio, lower power and smaller sizes could then be achieved. In this direction, suspended gas sensors appear to be the preferred architecture due to the low-power recovery and low noise performance.
